# Neuroprotective Effects of Melittin Against Cerebral Ischemia and Inflammatory Injury via Upregulation of MCPIP1 to Suppress NF-κB Activation In Vivo and In Vitro

**DOI:** 10.1007/s11064-023-04030-7

**Published:** 2023-10-09

**Authors:** Xing Xing, Xiangjian Zhang, Jingyi Fan, Cong Zhang, Lan Zhang, Ruisheng Duan, Hongyu Hao

**Affiliations:** 1 Present Address: Department of Neurology, Second Hospital of Hebei Medical University, Shijiazhuang, China; 2 Department of Neurology, Hebei General Hospital, Shijiazhuang, China; 3 Hebei Key Laboratory of Vascular Homeostasis and Hebei Collaborative Innovation Center for Cardio-Cerebrovascular Disease, Shijiazhuang, China

**Keywords:** Melittin, MCPIP1, Neuroprotection, Ischemic stroke, Neuroinflammation, NF-κB

## Abstract

Melittin, a principal constituent of honeybee venom, exhibits diverse biological effects, encompassing anti-inflammatory capabilities and neuroprotective actions against an array of neurological diseases. In this study, we probed the prospective protective influence of melittin on cerebral ischemia, focusing on its anti-inflammatory activity. Mechanistically, we explored whether monocyte chemotactic protein-induced protein 1 (MCPIP1, also known as ZC3H12A), a recently identified zinc-finger protein, played a role in melittin-mediated anti-inflammation and neuroprotection. Male C57/BL6 mice were subjected to distal middle cerebral artery occlusion to create a focal cerebral cortical ischemia model, with melittin administered intraperitoneally. We evaluated motor functions, brain infarct volume, cerebral blood flow, and inflammatory marker levels within brain tissue, employing quantitative real-time polymerase chain reaction, enzyme-linked immunosorbent assays, and western blotting. In vitro, an immortalized BV-2 microglia culture was stimulated with lipopolysaccharide (LPS) to establish an inflammatory cell model. Post-melittin exposure, cell viability, and cytokine expression were examined. MCPIP1 was silenced using siRNA in LPS-induced BV-2 cells, with the ensuing nuclear translocation of nuclear factor-κB assessed through cellular immunofluorescence. In vivo, melittin enhanced motor functions, diminished infarction, fostered blood flow restoration in ischemic brain regions, and markedly inhibited the expression of inflammatory cytokines (interleukin-1β, interleukin-6, tumor necrosis factor-α, and nuclear factor-κB). In vitro, melittin augmented MCPIP1 expression in LPS-induced BV-2 cells and ameliorated inflammation-induced cell death. The neuroprotective effect conferred by melittin was attenuated upon MCPIP1 knockdown. Our findings establish that melittin-induced tolerance to ischemic injury is intrinsically linked with its anti-inflammatory capacity. Moreover, MCPIP1 is, at the very least, partially implicated in this process.

## Introduction

Melittin, the major bioactive component (40–50%) [1] of honeybee (*A. melifera*) venom, is a hemolytic, small, linear peptide composed of 26 amino acid residues with the following sequence: GIGAVLKVLTTGLPALISWIKRKRQQ-NH2 [2]. It has a hydrophobic N-terminus and a hydrophilic C-terminus, forming channels on the plasma membrane [3]. Previous studies demonstrated that melittin has antibacterial [2], anti-inflammatory [4], anti-arthritis [5], anti-tumor [6], and neuroprotective properties [7–9]. The neuroprotective effects against neurological disorders mainly included enhancing motor performance [10] and protecting neurons, inhibiting oxidative stress and alleviating memory impairments [7], decreasing neuroinflammation [9], anticonvulsant potential [11], etc. Few studies have examined the potential application and benefit of melittin for stroke.

Stroke remains the leading cause of death and the most common cause of permanent disability worldwide, with ischemic stroke accounting for approximately 85% of all cases [12]. A key target of current ischemic stroke studies is inflammatory mechanisms that initiate within minutes after acute cerebral ischemia and persist for a long duration. Therefore, intervention against inflammation may be a prospective therapeutic target [13]. Neuroinflammation induced by neurocyte death after stroke triggers a cascade of events, including the overactivation of multiple cytokines and pathways [14], ultimately causing secondary injury and aggravating brain damage, and is integral to the pathophysiology of ischemic stroke [15]. In this process, nuclear factor-κB (NF-κB) and its pathways can be activated by oxidative stress [16], cerebral ischemia, and hypoxia, triggering a myriad of pro-inflammatory responses in microglia after brain ischemia, including upregulation of inflammasome components, such as tumor necrosis factor-alpha (TNF)-α, interleukin (IL)-6, IL-1β, which can further increase inflammatory damage [17].

Meanwhile, molecules with anti-inflammatory functions, such as tumor growth factor beta (TGF-β), IL-10, and IL-4, can counteract the effects of the aforementioned pro-inflammatory cytokines [15]. Monocyte chemotactic protein-induced protein 1 (MCPIP1, also known as ZC3H12A) is a recently identified zinc-finger protein that is a negative regulator of the inflammatory response [18]. Specifically, MCPIP1 inhibits MCP-1, IL-1β, IL-6, and TNF-α production by inhibiting the c-Jun N-terminal kinase and NF-κB signaling pathways [19]. Recently, researchers found that some agents could mediate neuroprotection during cerebral ischemia via MCPIP1 [20, 21], such as tetramethylpyrazine [22, 23], lipopolysaccharide (LPS) [24], and minocycline [25]. Additionally, studies showed that MCPIP1 was involved in electroacupuncture pretreatment-induced delayed brain ischemia tolerance [26].

The present study has demonstrated that melittin can inhibit the expression of inflammation markers (IL-1β, IL-6, TNF-α, interferon-gamma, and MCP-1) in the heart induced by coxsackievirus B3 [27]. Additionally, melittin administration effectively corrected the heightened levels of TNF-α and IL-6 while simultaneously suppressing upstream signaling molecules such as Toll-like receptor 4 (TLR4), p38 mitogen-activated protein kinase, and NF-κB in an acetic acid-induced colitis model [28]. Based on these findings, we hypothesize that melittin may also exert neuroprotective effects against cerebral ischemic conditions.

Recent In vivo and In vitro investigations have revealed that melittin conveys neuroprotective and organ-protective influences in neurodegenerative diseases, acting through anti-apoptotic and anti-inflammatory pathways by hindering the NF-κB signaling pathway [11, 28]. Furthermore, melittin has been observed to manifest anti-inflammatory properties in BV-2 microglia by diminishing levels of nitric oxide and inducible nitric oxide synthase, thus obstructing LPS-induced NF-κB activation [29]. Consequently, in this study, we evaluated whether melittin administration could offer protection against focal cerebral ischemia in an animal model employing distal middle cerebral artery occlusion (dMCAO). We also scrutinized the repression of melittin-induced regulation of microglial inflammasome activation within LPS-stimulated BV-2 cells. Mechanistically, we investigated the impact of melittin on NF-κB pathway inhibition and MCPIP1 upregulation. This novel insight into the MCPIP1-induced anti-inflammatory activity implies that melittin might be a pioneering therapeutic agent for ischemic stroke and potentially for other neuroinflammatory diseases.

## Materials and Methods

### Animals and Melittin Administration

Male, specific pathogen-free, C57/BL6 mice (20–25 g) were supplied by Vital River (Beijing Vital River Laboratory Animal Technology, Beijing, China). In all experiments, 8- to 12-week-old mice were used, housed in a controlled animal facility in Hebei Key Laboratory of Vascular Homeostasis, Second Hospital of Hebei Medical University. All mice were supplied with water and balanced nutritional rodent chow and housed in controlled conditions with a 12-h light/dark cycle and humidity of 60 ± 5% at 22 ± 3 °C. The experimental procedures were approved by the Experimental Animal Ethics Committee of Hebei Medical University (Shijiazhuang, China, Permit No. HMUSHC-130318). All studies were performed per the Guide for the Care and Use of Laboratory Animals (8th Edition) and the ARRIVE guidelines.

Melittin (purity 98%; measured using high-performance liquid chromatography; Xian Lintai Bioscience & Technology Co. Ltd., China) was diluted with 0.9% saline and administered intraperitoneally. In preliminary experiments, the median lethal dose (LD50) of melittin was less than 100 µg/g. Mice were randomly divided into the following groups: MEL groups: mice treated with a dose of 0.1 μg/g (MEL-L), 0.2 μg/g (MEL-M), or 0.4 μg/g (MEL-H) melittin 24 h before ischemia and once a day after surgery until sacrificed. Mice in the sham and Vehicle groups were intraperitoneally injected with an equal volume of 0.9% saline at the corresponding time points.

### Mouse Focal Brain Ischemia Model

Focal cerebral cortical ischemia was established by permanent occlusion of the unilateral middle cerebral artery (MCA) and common carotid artery (CCA), as described previously [30]. Weighed animals were anesthetized with an intraperitoneal injection of avertin (400 mg/kg, Cat# T48402-25G, Sigma-Aldrich, USA). The body temperature was monitored and maintained at 37.5 ± 0.5 °C. For dMCAO (Vehicle group), a median neck incision (approximately 1 cm) was performed, and the right CCA was isolated, exposed, and permanently ligated with a surgical suture. A skin incision was made between the right eye and the external auditory canal. Then the cortical branch of the right MCA was exposed by drilling a small hole, approximately 2 mm in diameter, through the skull. The MCA was then coagulated with a cauterizer (Bovie, USA) under a microscope to avoid damaging the brain surface. Sham-operated control mice underwent the same procedure except for CCA occlusion and distal MCA coagulation.

### Neurological Function Assessment

The rotarod test was performed to evaluate the motor coordination and learning function of ischemic mice per the procedures described by Hayashi-Takagi et al. [31]. The modified neurological severity score (mNSS) was determined to assess neurological function, including motor, sensory, reflex, and balance abilities, learning, and limb coordination skills, following the criteria reported by Gao et al. [32]. The mNSS scores were recorded at 24 h, 48 h, and 72 h after dMCAO, and grading was performed using a modified scale ranging from 0 to 18 (normal score, 0; maximal deficit score, 18). Higher scores suggested more severe neurological impairment. Mice that could remain on a fixed (4 rpm) rotating rod for at least 60 s were selected for the rotarod test and divided into four groups: Vehicle (dMCAO), MEL-L, MEL-M, and MEL-H (exact dosages as described above). After training for five days, the animals were placed on the rod with an accelerating speed from 4 to 40 rpm in 4 min for three trials at each time point, and then the results were averaged. Twelve male mice were used in each group.

### Brain Infarction and Water Content Measurement

The brains were stained with 2,3,5-triphenyltetrazolium chloride (TTC) to evaluate infarct volume at 24 h after dMCAO, as described previously. Mice were euthanized, and the brains were removed and frozen for 20 min. The frozen brain tissue was sectioned coronally at a thickness of 1 mm and incubated in 2% TTC at 37 °C for 15 min, followed by fixation in 4% paraformaldehyde for 24 h. TTC reacted with dehydrogenase in normal tissue and stained red, and ischemic tissue appeared pale because of its low dehydrogenase activity. The infarct volume was quantified using image analysis software (Image-Pro Plus 5.1; Media Cybernetics, Inc., Bethesda, MD, USA) and expressed as a percentage of the contralateral hemisphere. To evaluate brain edema after cerebral ischemia, all animals were anesthetized with 4% isoflurane, and brains were removed at 24 h, 48 h, and 72 h after dMCAO. Brain tissues were weighed before (wet weight) and after (dry weight) drying at 95 °C for 24 h. We used the following formula to calculate the brain water content (%): (wet weight − dry weight)/wet weight × 100. Six mice were used in each group.

### Cerebral Blood Flow (CBF) Assessment

CBF was monitored in real-time using a laser speckle contrast imager (PeriCam PSI System, Perimed, Sweden). Anesthetized mice with the skull exposed were fixed on a stereotactic apparatus while undergoing scans. The images were used to calculate the average perfusion level in infarcted areas and assess CBF fluctuations in both hemispheres. The body temperature of mice was kept at 37 ± 0.2 °C during the operation.

### Cell Culture and Transfections

The murine BV-2 microglia cell line obtained from the National Collection of Authenticated Cell Cultures (Shanghai, China) was cultured in DMEM (Gibco, USA) supplemented with 10% (v/v) fetal bovine serum (Gibco) and 1% penicillin/streptomycin at 37 °C in a humidified atmosphere containing 5% CO_2_. Cells were passed when they were approximately 80% confluent. According to the experimental conditions, cells were inoculated in a cell culture dish. After the cells adhered to the wall, a follow-up study was performed.

BV-2 cells were pretreated with 1 μg/ml LPS for 24 h for the cellular inflammation model. Because the safe concentration of melittin in BV-2 cells was less than 4 μg/ml, cells were pretreated with three doses of melittin (Diluted with culture medium) for 1 h, 0.5 μg/ml (MEL-L), 1 μg/ml (MEL-M), or 2 μg/ml (MEL-H), before treatment with LPS. BV-2 cells in the control group were not pretreated with melittin.

Transfections with fluorescent MCPIP1 siRNA (si-ZC3H12A, GenePharma, Shanghai, China) and control siRNA (si-Control, GenePharma) were performed using Lipofectamine RNAiMAX strictly according to the manufacturer’s instructions. The siRNA sequences used in the experiment were as follows: si-ZC3H12A: 5′-CCUGGACAACUUCCUUCGUAAGAAA-3′; si-Control: 5′-UUUCUUACGAAGGAAGUUGUCCAGG-3′. melittin (2 μg/ml) was added to the culture medium 6 h after siRNA transfection. After 1 h of melittin pretreatment, the cell culture medium was replaced with a medium containing both LPS and melittin, and the cells were incubated for 24 h in a 5% CO_2_ incubator at 37 °C for analysis. Cells transfected with si-Control were used as controls.

### Cell Viability Assay

The cell viability rate was determined using a Cell Counting Kit-8 (CCK-8, Dojindo, Japan). The cells were seeded in 96-well plates at a density of 2 × 10^5^/ml and incubated for 24 h before experimental treatments. At each time point, processing was ended, and 10 μl of CCK-8 was placed in each well. After incubation at 37 °C for 2 h, the absorbance at 450 nm was measured using a microplate reader (TECAN, Swiss). The cell viability of the test groups was expressed as the percentage of viable cells normalized to that of the control group. Six replicated wells were set up in each group. The results represent three independent experiments.

### Real-Time Polymerase Chain Reaction (RT-PCR)

Briefly, total RNA was extracted from ischemic brain tissues and BV-2 cells using TRIzol (Invitrogen, USA), isolated using a Total RNA Purification kit (Nanohelix, Daejeon, Korea) following the manufacturer’s instructions, and then reverse-transcribed to cDNA using a synthetic first chain cDNA toolkit (Fermentas International Inc.). Quantitative RT-PCR (qRT-PCR) was performed using a fluorescent dye with a LightCycler480 PCR instrument (SYBR Green I; Cwbio). Forty cycles were conducted as follows: 95 °C for 10 s, 60 °C for 20 s, and 72 °C for 20 s. The mRNA level was normalized to that of mouse GAPDH and expressed as the fold change. The mouse-specific primers (Sango Biotech, Shanghai, China) were as follows: IL-1β: F: ACTGTTTCTAATGCCTTCCC; R: TGGTTTCTTGTGACCCTGA, IL-6: F: TCCAGTTGCCTTCTTGGGAC; R: GTGTAATTGCCTCCGACTTG, TNF-α: F: CCAGTGTGGGAAGCTGTCTT; R: AAGCAAAAGAGGAGGCAACA, NF-κB: F: GGCTGTATTCCCCTCCATCG; R: CCAGTTGGTAACAATGCCATGT, MCPIP-1: F: CAATGTGGCCATGAGCCAT; R: AGTTCCCGAAGGATGTGCTG, MM-GAPDH: F: GGTTGTCTCCTGCGACTTCA; R: TGGTCCAGGGTTTCTTACTCC. In vivo, we obtained tissue samples from the brain at 24 h, 48 h, and 72 h after dMCAO. Six mice were used in each group at each time point. In vitro, six replicated wells were set up in each group.

### Enzyme-Linked Immunosorbent Assay (ELISA)

Brain homogenates were prepared using a tissue homogenizer, and the supernatant was collected for detection after centrifugation. For In vitro experiments, the cell culture supernatant after centrifugation was used for further studies. The Mouse IL-6 ELISA Kit (JEM-05, Anhui Joyee Biotechnics, China), Mouse IL-1β ELISA Kit (JEM-01, Anhui Joyee Biotechnics), and Mouse TNF-α ELISA kit (JEM-12, Anhui Joyee Biotechnics) were used to measure the protein concentration of tissue and cell IL-6, IL-1β, and TNF-α according to the manufacturer’s instructions. A microplate reader (Infinite M200 PRO, Tecan, Switzerland) was used for the analysis. Observation time points, grouping, and sample size are the same as above.

### Western Blotting

Total protein was extracted from brain tissues using a Total Protein Extraction Kit (Applygen Technologies Inc. Beijing, China) and a Nuclear Protein Extraction Kit (CWBIO, Beijing, China). Lysis buffer was prepared containing phenylmethylsulfonyl fluoride (Applygen Technologies Inc. Beijing, China), a protease inhibitor (Sigma, USA), and cell RIPA buffer (Solarbio, Beijing, China) with a ratio of 1:1:100. Adherent and centrifuged cells were separated, mixed with prepared lysis buffer, and lysed on ice for 30 min. After centrifugation at 4 °C and 12,000 × g for 20 min, the supernatant was assessed using a protein concentration assay. Nucleoprotein from brain tissues and cells was extracted using a Nuclear Protein Extraction Kit (CWBIO, Beijing, China) in strict accordance with the instructions. Protein concentrations were determined using a bicinchoninic acid protein assay reagent kit (Thermo Scientific, USA). Proteins (50 µg) were separated on a 10% sodium dodecyl sulfate–polyacrylamide gel and transferred onto polyvinylidene difluoride membranes (Roche, USA) in a transfer buffer containing 0.1% sodium dodecyl sulfate. The membranes were blocked with 5% skimmed milk for 1 h and incubated with primary antibodies consisting of mouse anti-β-actin (1:15,000, GeneTex, USA), mouse anti-MCPIP1 (1:1000, Abcam, US), rabbit anti-TLR4 (1:500, SAB), mouse anti-P84 (1:1000, GeneTex), and rabbit anti-NF-κB (1:500, Cell Signaling, USA) in blocking buffer overnight at 4 °C with gentle shaking. After three rinses (10 min each) with TPBS (phosphate-buffered saline (PBS) and 0.1% Tween-20), the membranes were incubated with secondary fluorescent antibodies (goat anti-rabbit or goat anti-mouse, 1:10,000; Rockland) at 37 °C for 1 h and then washed three times with TPBS (10 min each). An Odyssey infrared imaging system (LI-COR Bioscience) was used to scan and measure the relative density of target bands. The ratios of the protein bands of interest and the loading control (β–actin for total and cytoplasmic protein, P84 for nuclear protein) were calculated using Image-Pro Plus 5.1, and the data were normalized to those of the sham condition.

### Cellular Immunofluorescence Staining

The immunofluorescence technique was performed as described previously [24]. Cells were washed with PBS and fixed in fresh 4% paraformaldehyde solution for 30 min at room temperature. The cells were then washed three times with PBS, incubated for 30 min in a blocking solution with 10% donkey serum at room temperature, and then incubated with a specific primary monoclonal rabbit anti-NF-κB antibody (1:500, Cell Signaling, USA) diluted in blocking buffer (1:400) overnight at 4 °C in a humidified chamber. On the second day, chamber slides were washed three times with PBS and incubated for 1 h with the appropriate corresponding secondary antibody (Alexa Fluor 488 or 594, 1:800, Jackson Immuno Research) diluted in blocking buffer (1:500) at 20–37 °C. The cells were washed three times with PBS, incubated for 5 min with Hoechst (1:100) for nuclei staining at room temperature while protected from light, and mounted with Vectashield medium. Color images were acquired with a laser scanning confocal microscope (Zeiss LSM880, Germany), and 200 cells from each experiment were counted using ImageJ software.

### Statistical Analysis

All data are presented as the mean ± standard error of the mean. Pairwise comparisons between groups were analyzed using a T-test, and multiple comparisons were evaluated by one-way ANOVA followed by the least significant difference test. For all analyses, *P* < 0.05 was considered statistically significant.

## Results

### Melittin Ameliorates Neurological Deficits and Decreased CBF in dMCAO Mice

**Fig. 1:**
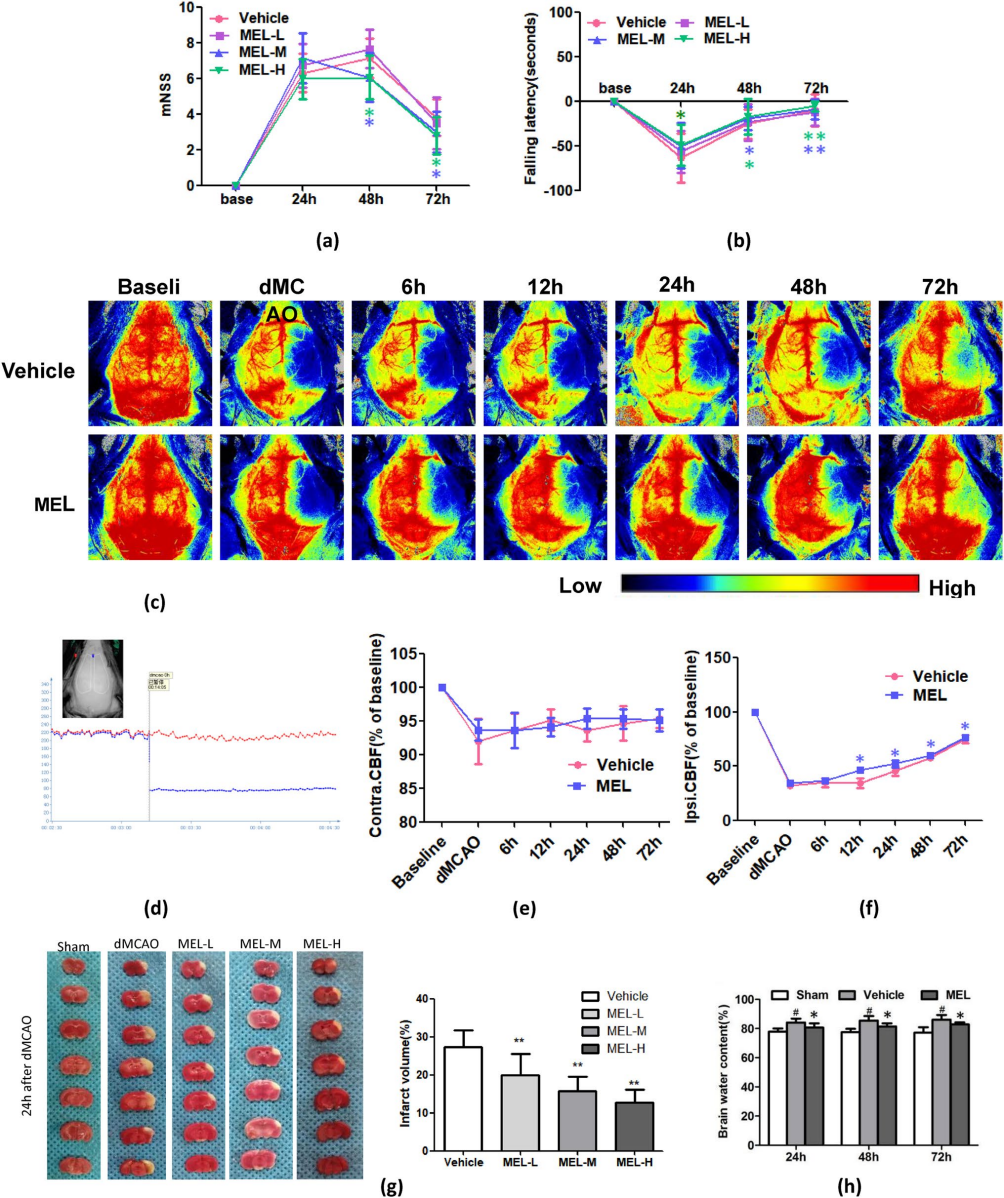
Melittin ameliorates neurological deficit and cerebral blood flow, reduces infarct volume and brain edema in dMCAO mice: **a** mNSS scores of dMCAO mice were significantly increased from 24 to 48 h and were reduced in medium- and high-dose of melittin-treated group than Vehicle group especially at 48 h and 72 h after operartion. **b** Neurologic impairment significantly recovered in middle- and high-dose melittin-treated mice at 24 h, 48 h and 72 h vs. Vehicle group. No significant difference in neurological deficits between low-dose MEL groups and Vehicle group. **c** Cerebral blood flow (CBF) was monitored in real time by laser speckle apparatus. The CBF decreased (blue) after dMCAO, but melittin (0.4 μg/g) could increase the CBF on the ischemic side compared to Vehicle group. **d** After dMCAO, the CBF of the operative side was significantly reduced than the contralateral side. **e** The CBF of the contralateral side decreased slightly. MEL group (0.4 μg/g) recovered slowly at 6 h, and there was no significant difference between the two groups. **f** The CBF of the lesion side decreased significantly immediately after dMCAO, and CBF was significantly recovered in MEL group from 12 to 72 h. **g** 24 h after operation, the brain tissue on the operation side of dMCAO appeared ischemic necrosis (gray). All MEL groups (0.4 μg/g) showed significant reduction in cerebral infarct size in a dose-dependent manner, compared with Vehicle group. **h** Brain edema on the lesion side significantly increases after dMCAO, especially at 72 h vs. Sham. Melittin (0.4 μg/g) decreased the percentage of brain water content in ipsilateral hemispheres after stroke at 24 h, 48 h and 72 h vs.Vehicle. *P* > 0.05, ∗*P* < 0.05, ∗∗*P* < 0.01 vs. Vehicle; #*P* < 0.05 vs. Sham

To assess the effects of melittin on neurological deficits following cerebral ischemia, dMCAO mice were pretreated with varying concentrations of melittin (0.1, 0.2, and 0.4 μg/g for the MEL-L, MEL-M, and MEL-H groups, respectively). The mNSS and rotarod test results were recorded at 24 h, 48 h, and 72 h post-operation intervals. The neurological scores in the MEL-M and MEL-H groups were notably lower than those in the vehicle group at both 48 and 72 h after dMCAO (*P* < 0.05) (Fig. 1a). Furthermore, the motor function test revealed significant recovery in neurologic impairment in the MEL-H group across all observation points, whereas no discernible difference in neurological deficits was found between the MEL-L group and the vehicle group (Fig. 1b). From these observations, we identified the effective therapeutic concentrations of melittin to be 0.2 μg/g and 0.4 μg/g. We consequently chose the medium dose (0.4 μg/g) for melittin administration in the subsequent experimental phase.

Bilateral CBF was carefully monitored using a laser speckle apparatus at specific time intervals: before the stroke and immediately, 6 h, 12 h, 24 h, 48 h, and 72 h afterward. The results indicated that CBF on the lesioned side sharply decreased after dMCAO. However, perfusion in the ischemic cortex of melittin-pretreated mice gradually increased from 12 to 72 h after stroke, showing a marked improvement compared to the vehicle group (Fig. 1c, d, f). CBF on the contralateral side in both vehicle and MEL groups began to recover slowly at 6 h post-stroke. However, no significant differences were observed between these two groups (Fig. 1e). These findings affirm that melittin enhances both motor deficits and CBF within the ischemic cortex in the dMCAO mouse model.

### Melittin Reduces Infarct Volume and Brain Edema in Ischemic Brain Injury

After dMCAO, the brain infarct size was assessed using TTC staining, which showed that the infarct size in melittin-pretreated brain tissue was reduced compared with that in the vehicle group at 24 h (*P* < 0.01) and it is dose-dependent (Fig. 1g). Brain edema is one of the earliest pathological processes after ischemic neuronal damage, and it significantly increases as early as 20 to 45 min after dMCAO and further increases over 72 h. Our results showed that melittin decreased the percentage of brain water content in the ipsilateral hemisphere after stroke at 24 h, 48 h, and 72 h compared with that in the vehicle group (P < 0.05) (Fig. 1h). These results indicated a potential effect of melittin in alleviating infarct volume and encephaledema after stroke In vivo.

### Melittin Inhibits Pro-inflammatory Factors and Induces MCPIP1 Expression in the Ischemic Brain

**Fig. 2:**
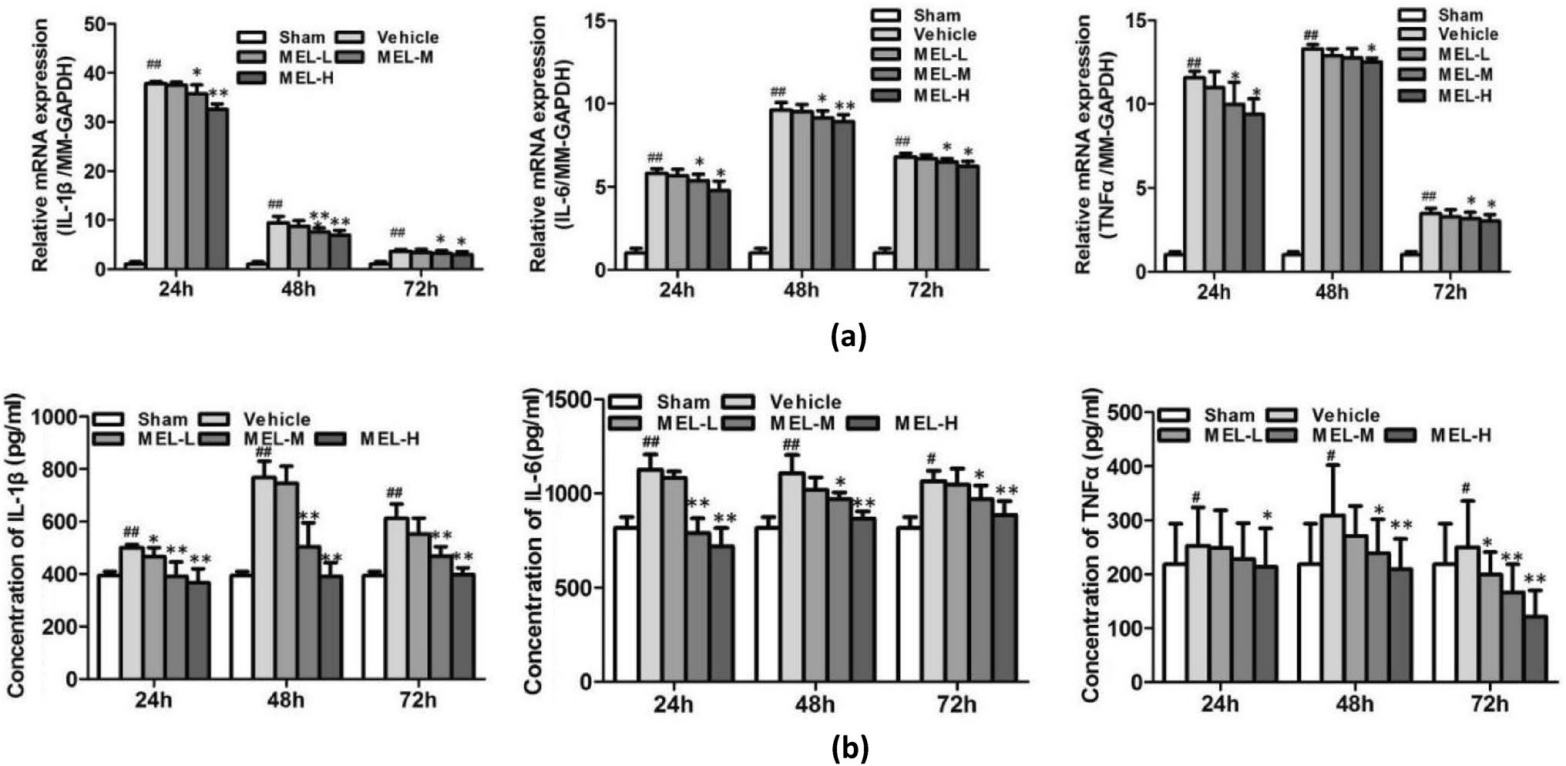
Melittin inhibits the expression of proinflammatory cytokine after dMCAO in ischemic brain tissue: **a** qRT-PCR was perfomed to assess the mRNA expression of IL-1β, IL-6, TNF-α in ischemic brain tissue at 24 h, 48 h, and 72 h after dMCAO. Vehucle droup showed significant increase of mRNA expression of inflammatory factors. Compared with Vehicle group, the medium and high dose MEL groups showed decreased mRNA levels of IL-1β, IL-6, and high dose MEL group showed decreased mRNA levels of TNF-α at each observation time point. **b** ELISA assay was performed to confirm the concentrations of IL-1β, IL-6, TNF-α in dMCAO mice. Consistent with the PCR results, ELISA results showed that the medium and high dose melittin pre-treatment reduced the the protein expression level of IL-1β and IL-6 at 24, 48, and 72 h. The protein concentration of TNF-α was downregulated by the high-dose MEL group at 24, 48, and 72 h. *P* > 0.05, ∗*P* < 0.05, ∗∗*P* < 0.01 vs. Vehicle; #*P* < 0.05, ##*P* < 0.01 vs. Sham.

**Fig. 3:**
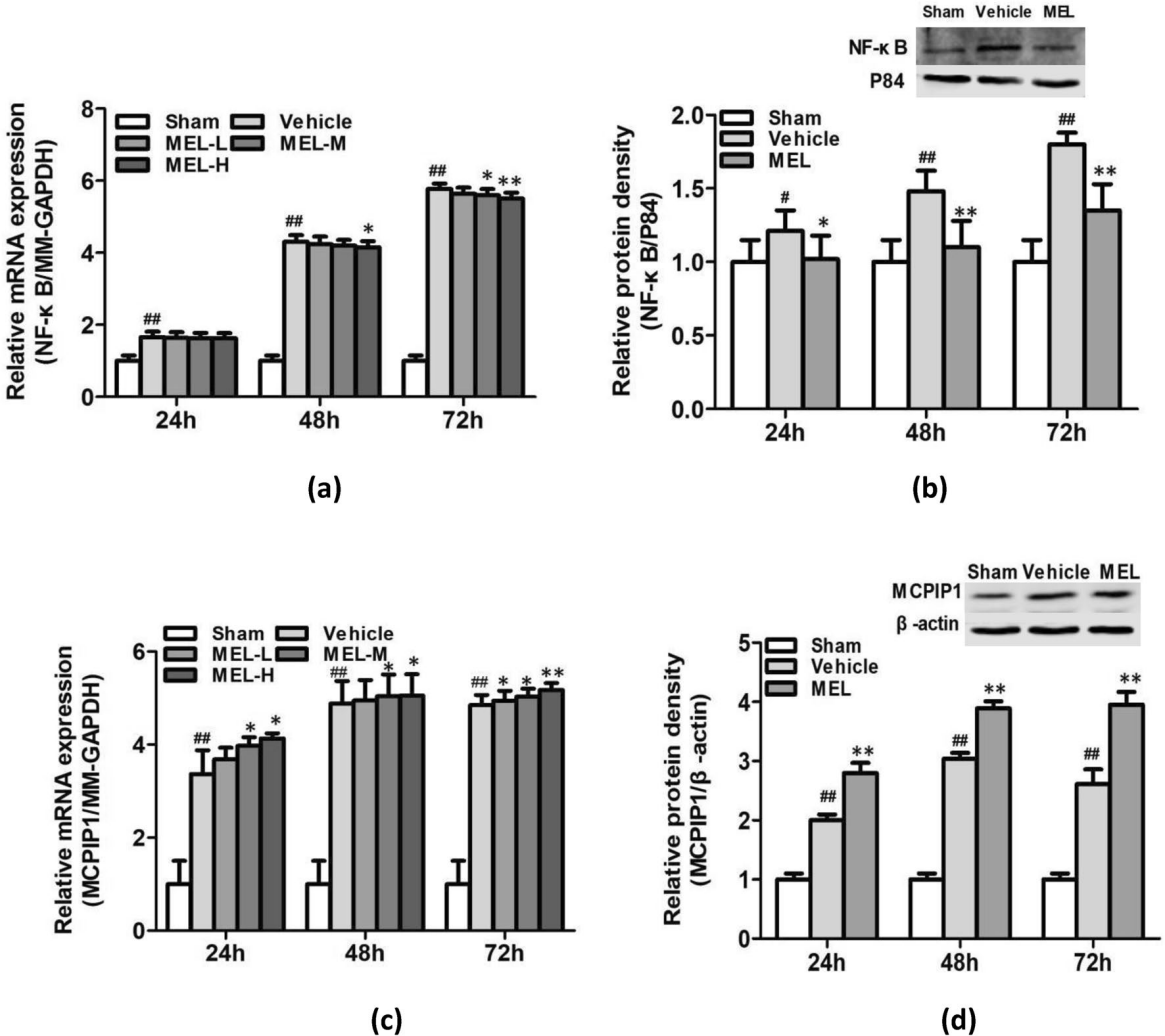
Melittin inhibits NF-κB and induces MCPIP1 expression: **a** qRT-PCR was performed to assess the mRNA expression of NF-κB in ischemic brain tissue. At 72 h after dMCAO, the mRNA in the MEL group was significantly downregulated compared to Vehicle group in the middle and high dose. **b** The western blot results showed the protein expression was decreased in MEL group (0.4 μg/g) compared to Vehicle group. **c** MCPIP1 mRNA level in ischemic brain tissue was increased after dMCAO compared with Sham group, the level peaked at 48 h and began to decline before 72 h. MCPIP1 mRNA expression in MEL-M and MEL-H group were steadily upregulated over 72 h. **d** The concentrations of MCPIP1 protein in ischemic brain tissues were determined by Western blot. The protein level of MCPIP1 was significantly increased in MEL group (0.4 μg/g) compared to Vehicle group. *P* > 0.05, ∗*P* < 0.05, ∗∗*P* < 0.01 vs. Vehicle; #*P* < 0.05, ##*P* < 0.01 vs. Sham

We examined the expression of pro-inflammatory cytokine transcripts in ischemic mouse brains after MCAO. The mRNA and protein levels of IL-1β, IL-6, and TNF-α in the ischemic brain were assessed by qRT-PCR and ELISA, respectively. Cerebral ischemia resulted in significantly increased levels of IL-1β, IL-6, and TNF-α compared with those after sham treatment. These increases were inhibited by melittin treatment in a dosage- and time-dependent manner (Fig. 2a, b). Because activation of the NF-κB signaling pathway is involved in producing inflammatory factors, we determined whether melittin pretreatment can affect this process. Compared with the vehicle group, NF-κB mRNA was significantly decreased 72 h after stroke in the MEL-M and MEL-H groups (Fig. 3a). The western blot results were consistent with the PCR results (Fig. 3b).

According to previous studies, MCPIP1 plays a significant anti-inflammatory role by inhibiting the generation of major pro-inflammatory cytokines [27]. Consistent with the previous findings, we found that the MCPIP1 mRNA level in ischemic brain tissue was slightly increased after dMCAO compared with that on the contralateral side; the level peaked at 48 h and began to decline before 72 h (*P* < 0.01) (Fig. 3c). Nevertheless, the qRT-PCR results indicated that MCPIP1 mRNA expression in the MEL-H group was steadily upregulated over 72 h (Fig. 3c). Additionally, the western blot results consistently showed that the protein level of MCPIP1 in ischemic brain tissue was significantly elevated by melittin treatment and was maintained at a high level 24–72 h after dMCAO (Fig. 3d). These findings indicate the potential of melittin to alleviate the neuroinflammatory injury induced by cerebral ischemia via inhibiting NF-κB and upregulating MCPIP1.

### Melittin Reduces LPS-Induced Cell Death and Inflammatory Cytokine Activation in BV-2 Cells

**Fig. 4:**
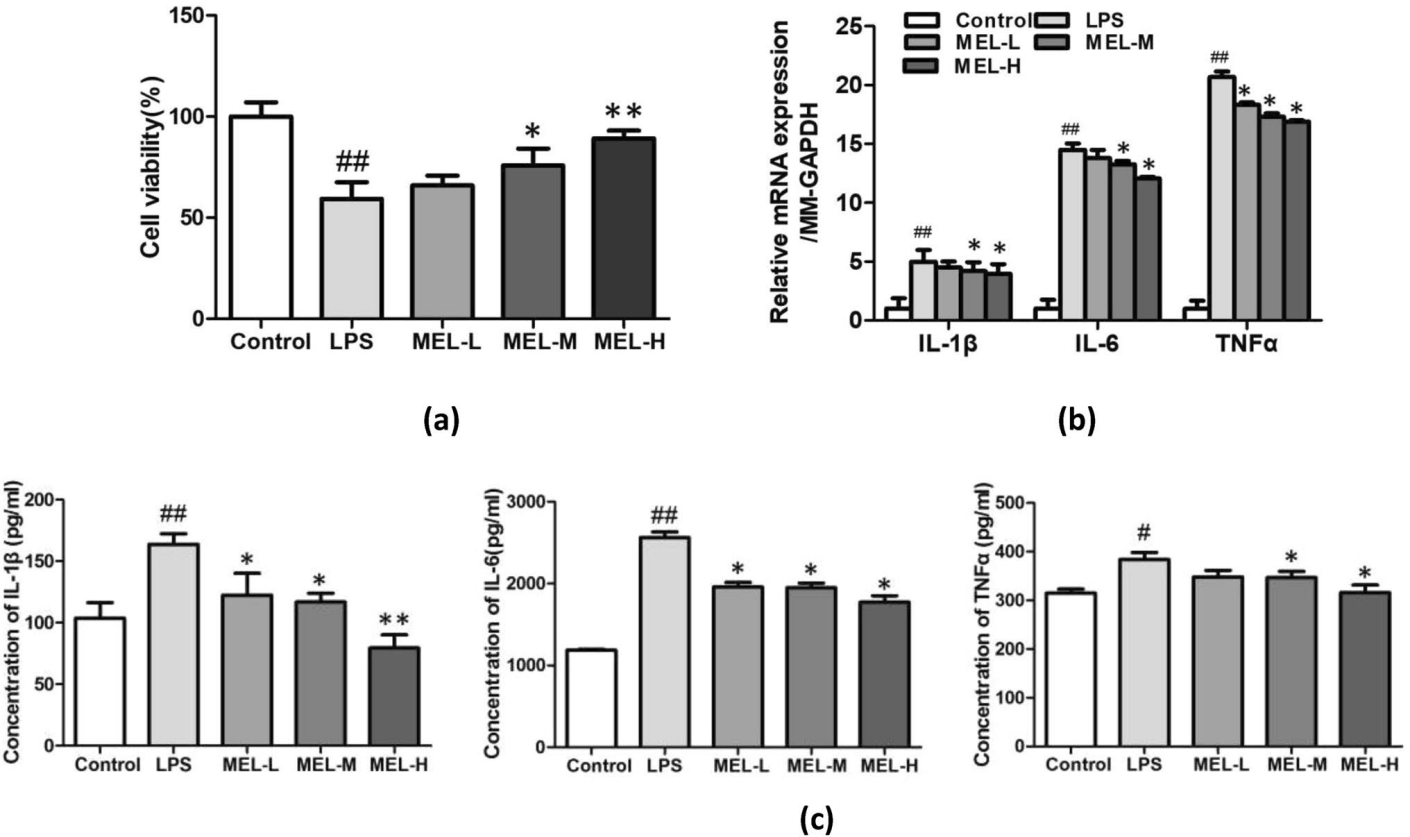
Melittin reduces LPS-induced cell death and inflammatory cytokine activation in BV-2: **a** CCK-8 results revealed that LPS stimulation of BV2 cells resulted in decreased cell viability and melittin pretreatment (1 μg/ml, 2 μg/ml) increased the survival. **b** The effects of melittin at different doses on the mRNA levels of IL-1β, IL-6, and TNF-α in LPS-treated BV-2 cells were assessed by qRT-PCR. Melittin of medium and high dose could apparently reduce IL-1β, IL-6, and TNF-αmRNA expression. **c** ELISA was performed to assess concentrations of IL-1β, IL-6, TNF-α protein, results showed protein level of these inflammatory factors significantly increased after LPS treatment,and reduced in medium and high dose of melittin group. *P* > 0.05, ∗*P* < 0.05, ∗∗*P* < 0.01 vs. LPS.; #*P* < 0.05, ##*P* < 0.01 vs. Control

We examined the protective effects of melittin against LPS-induced cell death in BV-2 cells. BV-2 cells were stimulated with LPS (1 μg/ml), incubated for 24 h, and pretreated with three doses of melittin for 1 h. Cell viability assays revealed that LPS stimulation of BV-2 cells resulted in decreased cell viability, and melittin pretreatment (1 μg/mL, 2 μg/mL) increased survival after LPS-induced inflammatory injury (Fig. 4a). To further clarify the mechanism of action, qRT-PCR and ELISA were performed to detect cytokine levels in LPS-induced BV-2 cells with or without melittin treatment. The results showed that melittin could apparently reduce IL-1β, IL-6, and TNF-α expression at the gene and protein levels with dose dependence in LPS-induced BV-2 cells (Fig. 4b, c). Combined with the results In vivo, the results In vitro indicated that the anti-inflammatory bioactivity of melittin participates in its neuroprotective effect against cerebral ischemia and neuroinflammatory injury.

### Melittin Suppresses Activation of NF-κB and Upregulates MCPIP1 Expression In vivo

**Fig. 5:**
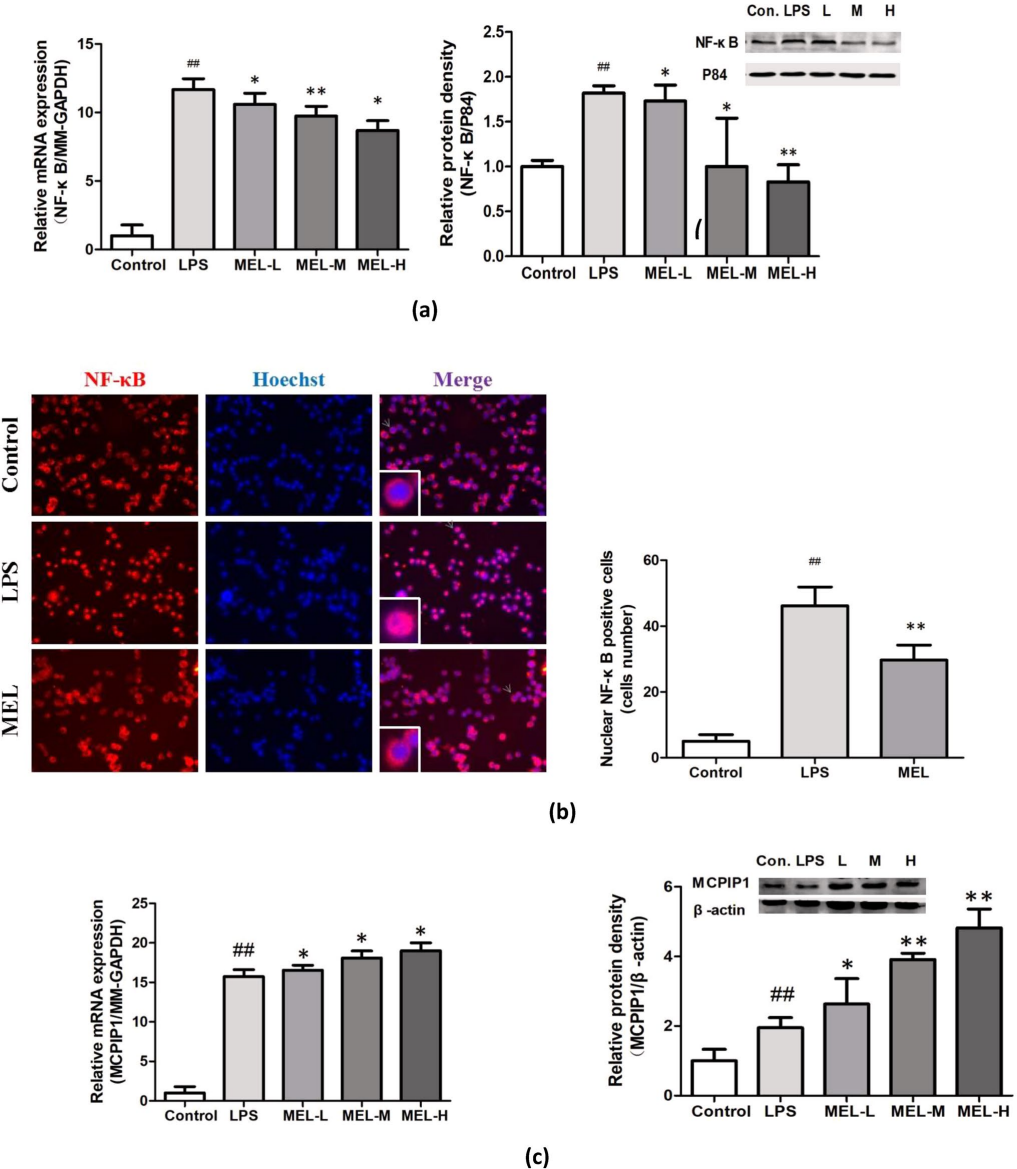
Melittin suppresses activation of NF-κB and upregulate expression of MCPIP1. **a** NF-κB mRNA and protein in LPS stimulated BV-2 cells were assessed by qRT-PCR and western blot. Results showed melittin groups with all dose presented a downregulation of NF-κB at both at mRNA and protein level. **b** Results of Cellular immunofluorescence staining: BV-2 cells were double-stained with anti-NF-κB (red) antibodies and Hoechst (blue) and the image merged. The NF-κB (p65 subunit) is mainly located in the cytoplasm when resting. Quantification of immunopositive BV-2 cells with anti-NF-κB in the nucleus presented the NF-κB pathway is activated and increases migration into the nucleus after LPS stimulation, and melittin pretreatment (2ug/ml) could inhibit the migratio.n of NF-κB to the nucleus. **c** qRT-PCR and western blot results showed MCPIP1 mRNA and protein in LPS stimulated BV-2 cells began to increase at 3 h, peaked at 6 h, and decreased at 12 h after LPS treatment, and were apparently elevated by melittin pretreatment in a dose-dependent manner. *P* > 0.05, ∗*P* < 0.05, ∗∗*P* < 0.01 vs. LPS.; #*P* < 0.05, ##*P* < 0.01 vs. Control

NF-κB plays a pivotal role in innate immune responses. Therefore, we investigated the effect of melittin on signaling molecules in LPS-induced BV-2 cells. The qRT-PCR and western blot results showed that melittin administration significantly ameliorated NF-κB expression at the mRNA and protein levels in BV-2 cells compared with those in the LPS group (Fig. 5a).

Moreover, the cellular immunofluorescence results showed that BV-2 cells were double-stained with anti-NF-κB (red) antibodies and Hoechst (blue), and the merged image indicated nuclear translocation. In the control group, most NF-κB was located in the cytoplasm. After LPS stimulation, NF-κB started to translocate into the nucleus. However, melittin treatment significantly inhibited LPS-induced nuclear translocation of NF-κB compared with the LPS group (Fig. 5b).

An investigation of anti-inflammatory factors showed that MCPIP1 mRNA increased at 3 h, peaked at 6 h, and decreased at 12 h due to LPS treatment compared with the control group (Fig. 5c). In LPS-stimulated BV-2 cells, the mRNA and protein levels of MCPIP1 were elevated by melittin pretreatment in a dose-dependent manner (Fig. 5c).

### Melittin Treatment-Induced Tolerance of Inflammatory Injury Decreases Because of MCPIP1 Deficiency in LPS-Induced BV-2 Cells

**Fig. 6:**
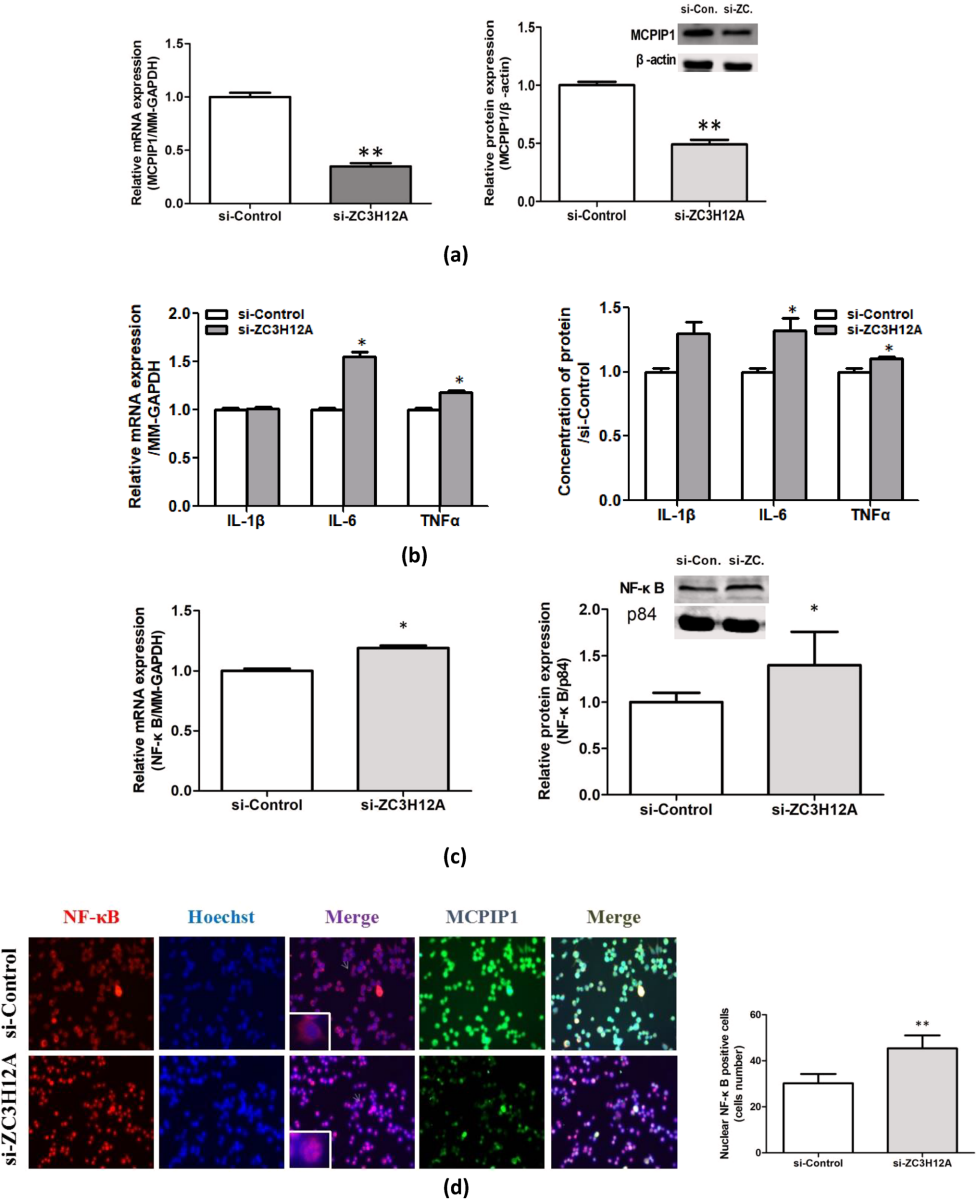
Loss of melittin-treatment-induced tolerance to inflammatory injury by MCPIP1 deficiency. **a** After transfection of Si-MCPIP1, MCPIP1 mRNA and protein expression were detected by PCR and Western blot, the results indicated that the expression decreased significantly after transfection, which proved that the transfection was successful. **b** qRT-PCR and ELISA results showed that MCPIP1 depletion significantly increased the expression of IL-1β, IL-6, TNF-α induced by LPS in BV-2 pretreated by melittin compared with si-Control. **c** The mRNA and protein levels of NF-κB according to the results of PCR and Western blot were marked elevation in si-ZC3H12A group. **d** When MCPIP was knocked down, the distribution of NF-κB in the nucleus was activated, even the BV-2 cells were treated by melittin. *P* > 0.05, ∗*P* < 0.05, ∗∗*P* < 0.01 vs. si-Control

Previous studies have shown that MCPIP1 may be a modulator that critically controls inflammation and immunity and alleviates inflammation by selectively suppressing the NF-κB pro-inflammatory signaling pathway [28]. Our previous results indicated that melittin treatment could increase MCPIP1 expression and reduce the NF-κB level. Consequently, a further study was conducted to examine whether MCPIP1 is involved in melittin treatment-induced neuroprotection against Inflammatory injury induced by LPS in BV-2 cells. After successfully knocking down MCPIP1 expression with MCPIP1-specific siRNA (Fig. 6a), BV-2 cells were treated with melittin and LPS, and the mRNA and protein expression levels of IL-1β, IL-6, and TNF-α were detected by qRT-PCR and ELISA. The results showed that MCPIP1 depletion significantly increased the expression of IL-1β, IL-6, and TNF-α induced by LPS in BV-2 cells pretreated with melittin compared with that of the si-Control group (Fig. 6b). Furthermore, the absence of MCPIP1 caused a marked elevation in the mRNA and protein levels of NF-κB according to the PCR and western blot results (Fig. 6c). Consistently, the laser confocal immunofluorescence microscopy results indicated that nuclear translocation of NF-κB caused by LPS-induced inflammatory injury was increased in the si-ZC3H12A group, even when both groups were pretreated with melittin (Fig. 6d). These results revealed that the anti-inflammatory effect of melittin was weakened by MCPIP1 knockdown. These results provide direct evidence that the neuroprotective effects of melittin against LPS-induced inflammatory injury are mediated, at least in part, by MCPIP1.

## Discussion

In traditional Chinese medicine, bee venom has long been used against chronic pain, skin diseases, arthritis, inflammation, and cancer [2]. As the bee venom peptide, melittin is hydrophobic and amphipathic, showing archetypal membrane activity [33]. It is toxic to both cells and tissues at a high enough concentration. Nevertheless, various exciting and potentially useful biological activities have been reported for melittin at low concentrations [3, 5], conjugated to proteins to other molecules, or formulated in nanoparticles and liposomes [2]. Recent experimental studies have shown that melittin can reduce excessive immune responses and provide a new alternative for controlling inflammatory diseases, including skin inflammation, neuroinflammation [7, 34], atherosclerosis, arthritis, and liver inflammation [35].

Melittin possesses neurophilic properties, with the toxic effect initially causing subcortical excitation and later inducing extensive inhibition in the cortex and subcortical structures [36, 37]. Moreover, melittin has demonstrated a remarkable analgesic effect, traversing the blood–brain barrier (BBB) and affecting the central nervous system, thereby expanding the pain threshold and reducing pain sensitivity [2, 38]. A recent study has shown that subtoxic concentrations of melittin can temporarily open the paracellular tight junctions of the BBB [2]. Another study has demonstrated that a 150-μL dose containing 3 μM of melittin significantly increases BBB permeability without causing significant toxicity or neurologic effects [39]. Additionally, the neuroprotective effect of melittin has been observed in Parkinson’s and Alzheimer’s disease after intraperitoneal injection administration [7]. As the pathophysiology of ischemic stroke includes encompassing inflammatory responses, oxidative stress, and cell death within the ischemic focal area [33, 34], we speculate that melittin could alleviate cerebral ischemic injury by inhibiting the inflammatory response within ischemic brain tissue and cells. By electrocoagulation, we established an experimental model of cerebral infarction, dMCAO and carried out a series of ethological, morphological, and molecular biology experiments. The In vivo results indicated that medium and high doses of melittin could significantly improve the motor function of mice with focal cerebral ischemia, reduce edema in brain tissue, decrease cerebral infarction volume, and promote blood flow recovery in ischemic brain tissue. These findings suggest that melittin has neuroprotective potential for preventing brain tissue injury caused by ischemia.

Neuroinflammation activated within hours after brain ischemia is a prime target for developing new stroke therapies [15, 40, 41]. During cerebral ischemia, the expression of both pro-inflammatory, as TNF-α, IL-1β, IL-6, and anti-inflammatory cytokines, MCPIP1, rapidly increases throughout the brain tissue [42, 43]. In the process, NF-κB, as an essential transcription factor, is activated by these cytokines (IL-1, IL-6, and TNF-α), regulates numerous genes, including TNF-α, IL-6, IL-1β, matrix metallopeptidase 9 [12, 44]. This vicious cycle expands the initial inflammatory response [45] and increases the detrimental effects of cerebral ischemia [46]. Our qRT-PCR, ELISA, and western blot results showed that melittin reduced the ischemia-induced increases in IL-1β, IL-6, and TNF-α and inhibited NF-κB expression in the nucleus In vivo. The findings revealed that melittin exhibits anti-inflammatory effects against cerebral ischemia, thereby exerting neuroprotective effects.

To determine the pharmaceutical characterization and possible mechanism of action, we constructed a neuroinflammation model in BV-2 microglial cells stimulated by LPS [47]. A recent study by Ran et al. [48] has shown that in MCAO mice, microglia displayed enhanced nuclear translocation of NF-κB p65 after surgery, accompanied by elevated levels of TLR4 protein (*P* < 0.001) and increased phosphorylation of IKBα and p65. MCPIP1 has emerged as a negative regulator of macrophage activation, which effectively inhibits the production of pro-inflammatory cytokines, including TNFα, IL-1β, IL-6, and MCP-1. Here, we found that the secretion of inflammatory cytokines, including IL-1β, IL-6, and TNF-α, could be significantly reduced, and MCPIP1 was elevated by melittin pretreatment. The mRNA and protein levels of NF-κB were reduced, and inflammatory injury-induced nuclear translocation of NF-κB was reversed. These results align with those of In vivo Tests.

MCPIP1 is an endogenous protein prominently expressed in the brain, primarily localized in neurons and microglia, which play crucial roles as primary sources of pro-inflammatory cytokines during ischemia. [24, 49]. As a negative regulator of macrophage activation, MCPIP1 exerts significant anti-inflammatory effects by inhibiting the production of a primary group of pro-inflammatory cytokines [50–52], such as MCP-1, IL-1β, IL-6, and TNF-α, via inhibition of the c-Jun N-terminal kinase and NF-κB signaling pathways [53–55]. Previous evidence has revealed that MCPIP1 expression is induced in LPS-stimulated monocytes, macrophages, and endothelial cells and is involved in LPS preconditioning-induced ischemic brain tolerance. Jian Liang et al. confirmed that MCPIP1 is involved in LPS preconditioning-induced ischemic stroke tolerance via its anti-inflammatory activities [24]. Jin et al. found that MCPIP1 deletion results in increased infarct volume and inflammatory gene expression in mice with transient MCAO [22]. Recently, some medications, including minocycline [25, 44], Tetramethylpyrazine [22, 23], and Huoluo Xiaoling Pellet have also been found to mediate neuroprotection during cerebral ischemia via MCPIP1 [53, 56]. In this study, we observed a significant and sustainable increase in MCPIP1 expression induced by melittin pretreatment both In vivo and In vitro. Furthermore, siRNA-mediated inhibition of MCPIP1 significantly increased the gene and protein expression levels of IL-1β, IL-6, and TNF-α in LPS-induced BV-2 cells treated with melittin and attenuated the melittin-induced neuroprotective effects. Western blot and laser confocal immunofluorescence microscopy results demonstrated that melittin suppresses the LPS-induced increase in NF-κB expression and nuclear localization, and this protective effect was weakened by MCPIP1 knockout in BV-2 cells. Our results also indirectly confirmed the significant inhibitory effect of MCPIP1 on NF-κB, consistent with previous studies’ results.

From these findings, we deduce that melittin can mitigate the injury resulting from ischemic stroke. This effect appears to be mediated, at least partially, by the inhibition of inflammatory cytokines and the NF-κB pathway through the upregulation of MCPIP1 in ischemic brain tissues and cells. Our study uncovers novel insights into the potential therapeutic application of melittin for treating cerebral ischemia.

In this research, we explored and substantiated the neuroprotective influence of melittin in animal models of cerebral ischemia and a cell model of neuroinflammation for the first time. However, certain limitations warrant further improvement. As compared with living cells extracted from brain tissues, immortal cell lines differ in biological characteristics and may not fully replicate the genuine In vivo environment. Moreover, considering that cell transfection may diminish cellular activities and influence the outcomes of subsequent drug treatment, additional studies employing animal experiments with gene knockout are required.

We also observed that the anti-inflammatory impact of melittin was diminished with MCPIP1 knockdown but not entirely nullified. This leads us to hypothesize that other factors or pathways might contribute to this mechanism. Another concern arises from the poor stability of peptide drugs In vivo and the inherent toxicity of melittin. Drug administration was confined to small doses of the melittin monomer, as the detrimental effects of larger quantities, such as hemolysis, have limited its clinical applicability. Future research must address these challenges and may include experimental refinements or alterations to the molecular structure of the medication to expand its potential therapeutic scope [57, 58].

## Conclusion

Our findings furnish compelling evidence that underscores the advantageous role of melittin in the therapeutic intervention of ischemic stroke, with potential applications to other neuroinflammatory diseases as well. As for the underlying mechanism, our study illuminates that melittin combats inflammatory injury in ischemic brain tissues and ameliorates cell death in LPS-induced BV-2 cells. This effect is seemingly achieved through the dual action of reducing pro-inflammatory cytokines IL-1β, IL-6, and TNF-α, and augmenting MCPIP1, thereby attenuating the NF-κB pathway. We further discovered that the induction of MCPIP1 mediates the melittin-induced resilience to inflammatory damage and plays a vital role in the tolerance to brain ischemia induced by melittin pretreatment.

These insights position melittin as a promising candidate in the arsenal against neuroinflammatory disorders. However, the pathway to clinical application is intricate and necessitates further experimental and clinical investigation to substantiate its therapeutic potency and delineate its precise mechanisms of action.

## Data Availability

All datasets generated or analyzed during this study are included in this article.
